# Discovery of a novel natural compound, vitekwangin B, with ANO1 protein reduction properties and anticancer potential

**DOI:** 10.3389/fphar.2024.1382787

**Published:** 2024-04-03

**Authors:** Yohan Seo, Sion Lee, Minuk Kim, Dongguk Kim, Sung Baek Jeong, Raju Das, Armin Sultana, SeonJu Park, Nguyen Xuan Nhiem, Phan Thi Thanh Huong, Oh-Bin Kwon, Wan Namkung, Joohan Woo

**Affiliations:** ^1^ New Drug Development Center, Daegu Gyeongbuk Medical Innovation Foundation (KMEDIhub), Daegu, Republic of Korea; ^2^ Department of Medical Device Development Center, Daegu-Gyeongbuk Medical Innovation Foundation (KMEDI Hub), Daegu, Republic of Korea; ^3^ Department of Physiology, Dongguk University College of Medicine, Gyeongju, Republic of Korea; ^4^ Metropolitan Seoul Center, Korea Basic Science Institute (KBSI), Seoul, Republic of Korea; ^5^ Institute of Marine and Biochemistry, Vietnam Academy of Science and Technology (VAST), Hanoi, Vietnam; ^6^ College of Pharmacy, Yonsei Institute of Pharmaceutical Science, Yonsei University, Incheon, Republic of Korea; ^7^ Channelopathy Research Center (CRC), Dongguk University College of Medicine, Goyang, Gyeonggi-do, Republic of Korea

**Keywords:** anoctamin 1, vitekwangin B, prostate cancer, lung cancer, protein reduction, apoptosis

## Abstract

**Background:** Prostate cancer and non-small cell lung cancer (NSCLC) present significant challenges in the development of effective therapeutic strategies. Hormone therapies for prostate cancer target androgen receptors and prostate-specific antigen markers. However, treatment options for prostatic small-cell neuroendocrine carcinoma are limited. NSCLC, on the other hand, is primarily treated with epidermal growth factor receptor (EGFR) tyrosine kinase inhibitors but exhibits resistance. This study explored a novel therapeutic approach by investigating the potential anticancer properties of vitekwangin B, a natural compound derived from *Vitex trifolia*.

**Methods:** Vitekwangin B was chromatographically isolated from the fruits of *V. trifolia*. ANO1 protein levels in prostate cancer and NSCLC cells were verified and evaluated again after vitekwangin B treatment.

**Results:** Vitekwangin B did not inhibit anoctamin1 (ANO1) channel function but significantly reduced ANO1 protein levels. These results demonstrate that vitekwangin B effectively inhibited cancer cell viability and induced apoptosis in prostate cancer and NSCLC cells. Moreover, it exhibited minimal toxicity to liver cells and did not affect hERG channel activity, making it a promising candidate for further development as an anticancer drug.

**Conclusion:** Vitekwangin B may offer a new direction for cancer therapy by targeting ANO1 protein, potentially improving treatment outcomes in patients with prostate cancer and NSCLC. Further research is needed to explore its full potential and overcome existing drug resistance challenges.

## 1 Introduction

Prostate cancer is characterized by the overexpression of androgen receptors and prostate-specific antigen markers (Tan et al., 2015), which significantly influences its pathogenesis. Hence, hormone therapies for prostate cancer primarily target these markers (Tan et al., 2015; Dai et al., 2017). However, patients with prostatic small cell neuroendocrine carcinoma encounter treatment limitations, particularly with hormone therapy, because this type of cancer does not express androgen receptors or prostate-specific antigen markers (Shafi et al., 2013; Tan et al., 2015; Dai et al., 2017).

Non-small cell lung cancer (NSCLC) is a major subtype of lung cancer, constituting approximately 85% of all lung cancer cases (Imyanitov et al., 2021). Despite considerable progress in NSCLC diagnosis and therapy, the five-year survival rate remains low (Hirsch et al., 2017). The growth and survival of NSCLC cells rely on epidermal growth factor receptor (EGFR) signaling (Wu and Shih, 2018). EGFR tyrosine kinase inhibitors, including gefitinib, have been adopted as the first-line treatment for NSCLC (Westover et al., 2018). Unfortunately, nearly one-third of patients exhibit resistance initially, and those who are responsive may eventually develop resistance (Westover et al., 2018; Wu and Shih, 2018; Kohsaka et al., 2019). Therefore, there is an urgent need to identify novel therapeutic targets and develop innovative agents for treating prostate cancer and NSCLC.

Anoctamin 1 (ANO1), also known as transmembrane protein 16A, is a calcium-activated chloride channel that is upregulated in various cancer cells and contributes to cancer progression (Bill and Gaither, 2017). Numerous *in vitro* and *in vivo* studies have shown that pharmacological downregulation of ANO1 significantly inhibits cancer cell proliferation, growth, and migration (Zhong et al., 2021). Notably, our previous studies revealed high ANO1 expression in PC3 and PC9 NSCLC cells (Seo et al., 2017; 2021). Moreover, the pharmacological inhibition of ANO1 using diethylstilbestrol, luteolin, and other inhibitors affects both its channel function and protein levels and thus suppresses cancer cell proliferation and migration (Seo et al., 2017; 2021). Notably, some ANO1 functional inhibitors impede ANO1-mediated chloride secretion, thereby inducing side effects such as hypotension, constipation, and dry mouth syndrome (Namkung et al., 2011; Jang and Oh, 2014; Wang et al., 2021). These findings imply that reducing ANO1 protein levels without rapidly impairing ANO1 channel function could be advantageous for cancer treatment. To address this, we used high-throughput screening to identify vitekwangin B, a natural product that gradually decreases ANO1 protein levels.

*Vitex trifolia*, a small wild shrub that is extensively distributed in the northern mountains of Vietnam, has traditionally been employed in the treatment of various ailments, including liver disorders, tumors, rheumatic pain, inflammation, and sprains (Li et al., 2005; Shukri and Hasan, 2021). This plant is renowned for its multifaceted medicinal properties, including larvicidal, wound-healing, anti-HIV, anticancer, trypanocidal, antimicrobial, and antipyretic effects (Kiuchi et al., 2004; Li et al., 2005; Shukri and Hasan, 2021). Among the phytochemicals present in this plant, flavonoids, such as casticin (vitexicarpin), persicogenin, and penduletin, induce apoptosis in cancer cell lines by inhibiting the G2/M phase of the cell cycle (Li et al., 2005).

Vitekwangin B, a lignan previously isolated from *V. kwangsiensia* and *V. negundo* (Shen et al., 2019; Kamal et al., 2022), has been reported to have minimal inhibitory effects on nitric oxide production (Shen et al., 2019; Kamal et al., 2022). However, its potential anticancer effects via the regulation of other molecular targets, including ion channels, remain unknown. Therefore, we isolated vitekwangin B from *V. trifolia* fruit for the first time and investigated the potential pharmacological effects of vitekwangin B extracted from *V. trifolia*, with a primary focus on the inhibition of ANO1 and its physiological impact on prostate and NSCLC cancer cells. Through a comprehensive investigation, we aimed to determine the anticancer properties of vitekwangin B.

## 2 Materials and methods

### 2.1 Plant material

The fruits of *V. trifolia* L. were collected from the Xuan Thuy National Garden, Namdinh, Vietnam, in October 2021. Taxonomic identification was conducted by Dr. Nguyen The Cuong from the Institute of Ecology and Biological Resources, Vietnam Academy of Science and Technology (VAST). A voucher specimen (No. NCCT-P133) was submitted to the Institute of Chemistry at VAST.

### 2.2 Extraction and isolation

Dried *V. trifolia* L. fruit powder (5 kg) was subjected to methanol-based sonication (three times, each with 15 L MeOH). After solvent evaporation, the resulting methanol extract (150 g) was reconstituted in water and subsequently partitioned with *n*-hexane (H), CH_2_Cl_2_ (D), and EtOAc (E) to yield the H (30 g), D (7 g), and E (5 g) fractions, and an aqueous layer.

Fraction D was initially subjected to silica gel column chromatography using a stepwise hexane/acetone gradient, yielding seven sub-fractions (D1–D7). Subsequently, Fraction D2 was chromatographed on a YMC RP-18 column using a 1:1 v/v mixture of MeOH and water, leading to the isolation of D2Ⅰ (0.8 g). D2Ⅰ was further subjected to silica gel column chromatography using a hexane and acetone (10:1 v/v) mixture to yield D2Ⅰ1 (0.1 g). Finally, prep-HPLC employing a J’sphere ODS H-80 column (250 mm × 20 mm) was performed with a mobile phase of 25% aqueous acetonitrile at a flow rate of 3 mL/min to isolate vitekwangin B (15 mg).

### 2.3 Cell culture

PC9 cells stably expressing ANO1 and yellow fluorescent protein (YFP) were established as previously described (Jeong et al., 2022). Additionally, PC9 (an NSCLC cell line), PC3 (a human prostate cancer cell line), and HT29 (a human colon cancer cell line) cells were cultured in RPMI 1640. CHO cells were maintained in Dulbecco’s modified Eagle’s medium. All culture media were supplemented with 10% fetal bovine serum, 2 mM L-glutamine, 100 U/mL penicillin, and 100 μg/mL streptomycin.

### 2.4 Construction of ANO1-knockout (KO) cells

PLentiCRISPRv2 vector containing Cas9 and CRISPR guide RNA targeting ANO1 (CCT​GAT​GCC​GAG​TGC​AAG​TA; clone ID: X35909) was procured from GenScript (Piscataway, NJ, United States). A co-transfection of 1,500 ng CRISPR plasmid, 1,200 ng packaging plasmid (psPAX2), and 400 ng envelope plasmid (pMD2.G) was performed in HEK293T cells cultured in 6-well plates. After 48 h, the supernatant, which contained lentiviral particles, was filtered using a 0.45-μm syringe filter. Subsequently, the cells were treated with lentiviral particles mixed with fresh medium in a 1:1 ratio in 24-well plates. ANO1-KO cells were selected using puromycin (Sigma-Aldrich, St. Louis, MO, United States) 72 h after viral transduction.

### 2.5 Yellow fluorescent protein fluorescence quenching analysis

PC9 cells stably expressing the YFP variant (YFP-H148Q/I152L/F46L) and endogenous ANO1 were seeded into 96-well plates at a density of 5 × 10^3^ cells/well. After 48 h of incubation, the cells were washed twice with phosphate-buffered saline and incubated for 10 min with the test compounds dissolved in phosphate-buffered saline. YFP fluorescence was measured every 0.4 s for 5 s using the FLUOstar^®^ Omega microplate reader (BMG Labtech, Ortenberg, Germany). ANO1-mediated iodide influx was measured 1 s after injecting 100 μL of 70 mM iodide solution with 100 μM adenosine triphosphate (ATP) into each well. The inhibitory effects of the test compounds on ANO1 activity were assessed based on the initial iodide influx rate, which was calculated from the initial slope of the decrease in fluorescence following ATP injection.

### 2.6 Measurement of intracellular calcium levels

PC9 cells were cultured in black-walled 96-well microplates and loaded with Fluo-4 NW (Invitrogen, Carlsbad, CA, United States) according to the manufacturer’s protocol. After 1 h of incubation, the plates were transferred to a FLUOstar^®^ Omega microplate reader with custom Fluo-4 excitation/emission filters (485/538 nm) and Fluo-4 fluorescence was assessed.

### 2.7 Western blot analysis

Protein samples (20–60 μg) were separated using 4%–12% Tris-Glycine-PAG Pre-Cast Gel (Koma Biotech, South Korea) and transferred onto polyvinylidene fluoride membranes. Blocking was performed for 1 h using 5% bovine serum albumin in Tris-buffered saline containing 0.1% Tween 20. The membranes were incubated with primary antibodies, including anti-ANO1 (Abcam, United Kingdom) and anti-β-actin (Santa Cruz Biotechnology, Dallas, TX, United States), followed by horseradish peroxidase-conjugated anti-secondary IgG antibodies (Enzo Life Sciences, Inc., Farmingdale, NY, United States) for 1 h. Visualization was performed using the ECL Plus Western Blotting System (GE Healthcare, Chicago, IL, United States).

### 2.8 Cell viability assay

Cell viability assay was performed using the Cell Counting Kit CCK -8 (Dojindo, Rockville, MD, United States) and MTS cell viability assay was performed using CellTiter 96^®^ Aqueous One Solution Cell Proliferation Assay Kit (Promega, Madison, WI, United States). PC3, PC9, and HT29 cells were cultured in 96-well plates for 24 h. Once the cells reached approximately 20% confluence, vitekwangin B (0.03–300 μM) or vehicle (dimethyl sulfoxide) were added to the medium. After 48 h of treatment, the medium was completely removed, and CCK-8 or MTS assay was performed according to the manufacturer’s instructions. The absorbance of formazan was measured at a wavelength of 490 or 450 nm using a microplate reader (Synergy™ Neo, BioTek, Winooski, VT, United States).

### 2.9 Molecular docking analysis

Molecular docking predicts the preferred orientation of one molecule relative to another when bound to form a stable complex (Meng et al., 2011). The Glide XP docking module was employed in this study to provide structural details of the protein–ligand complex. Ligand preparation, protein preparation, active site determination, and grid generation were sequentially performed using Schrödinger Suite 2017-1 software (Adebesin et al., 2022). The ligands selected for analysis were obtained in an SDF file format from the PubChem database (Adebesin et al., 2022) and processed with the LigPrep module to ensure accurate 3D conformation at a low state and proper chiral position, maintaining pH 7.0 ± 2.0. The protein of interest was retrieved from the Protein Data Bank and subjected to a protein preparation wizard that optimized the bond angles, bond orders, and topology to generate the best possible structure. Irrelevant water was removed from the active site at 5 Å, and the root-mean-square deviation for heavy atoms was fixed at 0.30 Å. A cubic box was built around the reference ligand during grid generation to define the active sites. The van der Waals scaling factor and charge cutoff were set to 1.00 and 0.25, respectively. The entire process was followed by the all-atom OPLS3 force field (Das et al., 2023).

Glide offers three tiers of scoring functions: Glide-HTVS, Glide-SP (Standard Precision), and Glide-XP (Extra Precision). These scoring functions employ the Emodel scoring function, which primarily consists of the Glide-score and the protein–ligand coulomb–vdW energy. We chose Glide-XP docking for maximum accuracy, in which ligand sampling was performed in a flexible manner. The scaling factor and partial charge for nonpolar parts of the ligand were set at 0.80 and 0.15, respectively. After final docking, the complex with the best binding position was selected for further analysis.

### 2.10 Prime molecular mechanics with generalised born and surface area solvation (MM-GBSA)

MM-GBSA analysis of the prime module was performed on the complexes formed by the docking simulation. Using the OPLS3 molecular mechanics force field, MM-GBSA (Rastelli et al., 2010) calculates the relative binding energy by combining the molecular mechanics energies (EMM), an SGB solvation model for polar solvation (GSGB), and a nonpolar solvation term (GNP) composed of a non-polar solvent-accessible surface area and van der Waals interactions.

The total free energy of binding:

$$\Delta Gbind=Gcomplex-\left(Gprotein+Gligand\right)$$

where G=EMM+GSGB+GNP

### 2.11 Caspase-3/CPP32 colorimetric assay

Caspase-3/CPP32 colorimetric assay was performed according to the manufacturer’s instructions (#K106; Biovison, Milpitas, CA, United States). PC3 and PC9 cells were cultured in 6-well plates until they reached 70% confluence, and the test compound or Ani9 (an ANO1 inhibitor) was then added in the wells. After incubation for 24 h, 5 × 10^6^ cells were lysed in cell lysis buffer for 10 min at 4°C. The cells were centrifuged for 10 min, and the supernatants were collected. Thereafter, 100 μg protein or 50 μL buffer was added to each well with 2× reaction buffer containing 10 mM DTT. To measure caspase-3 activity, 5 μL DEVD-pNA substrate was added and incubated for 1 h at 37°C. The optical density was measured at the wavelength of 400 nm using a microplate reader (Synergy™ Neo).

### 2.12 Human cleaved PARP-1 activity assay

The human cleaved PARP-1 activity assay was performed according to the manufacturer’s instructions (#ab174441; Abcam). PC3 and PC9 cells were cultured in 6-well plates until they reached 80% confluence. The test compounds were then added and incubated with the cells for 24 h. Subsequently, 5 × 10^7^ cells were lysed in cell extraction buffer for 20 min. Supernatants were collected after the cells were centrifuged (13,000 RPM) for 20 min at 4°C. Next, 100 μg protein or 50 μL buffer was added to an antibody cocktail containing capture and detector antibodies in each well and incubated for 1 h. The wells were washed twice with 1× wash buffer. TMB development solution was added and the contents of the wells were incubated for 10 min. Finally, the stop solution was added and the optical density was measured at the wavelength of 450 nm using a microplate reader (Synergy^TM^ Neo).

### 2.13 Cell cycle analysis

PC9 cells were seeded at 2 × 10^5^ cells/well in 100-phi culture plates and treated with the test compound for 24 h. Cells were fixed with cold 70% ethanol and incubated for 2 h at −20°C. The ethanol was removed, and the cells were re-suspended in propidium iodide, Triton X-100, or DNAse-free RNAse A staining solution for 30 min. The cell cycle distribution was analyzed using Gallios (Beckman Coulter, Brea, CA, United States). Approximately 2 × 10^3^ cells per group were analyzed.

### 2.14 hERG inhibition assay by patch-clamping electrophysiology

To assess the cardiotoxic effects of the tested hERG channel-dependent chemical compounds, we measured the rate of hERG channel inhibition in an hERG-overexpressing HEK cell line (Eurofins Scientific, Luxembourg) using the PatchLiner automated patch-clamping system (Nanion, Germany).

The cells were incubated at 37°C under 5% CO_2_. Subsequently, the cells were detached from the cell culture plates using trypsin (SH30042.02; HyClone, Logan, UT, United States) and centrifuged at 200 × g. After washing with external standard buffer solution (08 3001; Nanion), the cells were resuspended in 5 mL of fresh external standard buffer solution. The resulting cell suspension was loaded into a PatchLiner system. The cells were then automatically dispensed into the wells of an NPC-16 chip (071102; Nanion) and one cell per well was sealed in a microhole at the bottom. Once a GΩ seal was formed, light and short suction pulses were applied to break through the membrane and establish whole-cell mode to electrically connect the cell with the Internal KF110 buffer solution (08 3007; Nanion).

To generate voltage stimulation specific to the hERG channel, the membrane potential of the cells was initially maintained at −80 mV, sequentially followed by −40 mV (0.5 s), +40 mV (0.5 s), −40 mV (0.5 s), and −80 mV (0.2 s) to generate hERG tail currents. The peak hERG tail current recorded from each well represented the baseline level of hERG channel activity. To investigate the changes in the hERG activity caused by the tested chemical compounds, the relevant chemical stock solutions were diluted with an external standard buffer solution to achieve the desired concentrations, which were automatically added to the wells of the NPC-16 chip. The hERG channel activity was then measured under the same voltage conditions. Finally, the relative changes in hERG activity induced by the tested chemical compounds were calculated and expressed as percentages using the following formula:

$$\%hERG\;activity=\left(peak\;hERG\;tail\;current\;after\;compound\;treatment/peak\;hERG\;tail\;current\;before\;compound\;treatment\right)\times 100$$

### 2.15 Resazurin reduction assay for evaluating hepatocellular viability

Hepatocellular viability was assessed using a resazurin reduction assay. HepG2 hepatocellular carcinoma cells (10,000 cells) were seeded in each well of a black and clear-bottom 96-well plate, with Dulbecco’s modified Eagle’s medium High glucose media supplemented with 10% (v/v) fetal bovine serum and 100 μg/mL penicillin–streptomycin. The cells were then incubated in a 5% CO_2_-supplied 37°C incubator for 15–20 h.

The following day, cells were treated with the tested compounds at four different concentrations (0, 0.05, 0.5, 5, and 50 μM) using Dulbecco’s modified Eagle’s medium High glucose media supplemented with 1% (v/v) fetal bovine serum and 100 μg/mL penicillin–streptomycin. For the maximum and minimum cell viability control experiments, 0.5% (v/v) dimethyl sulfoxide solvent and 0.01% (v/v) Triton X-100 were used, respectively.

After incubation for 20 h, each well was treated with 20% (v/v) resazurin reagent (#G8080; Promega) and further incubated for 2 h. Colorimetric analysis of resorufin levels in each well was performed by detecting the fluorescence intensity at the wavelength of 590 nm. Each data point was normalized using the maximum/minimum cell viability data and presented as “%Relative viability” using the following formula:

$$\%Relative\;viability=\left(experimental\;datum\;–\;minimal\;viability\;datum\right)/\left(maximal\;viability\;datum\;–\;minimal\;viability\;datum\right)\times 100$$

### 2.16 Statistical analysis

All experiments were conducted independently at least three times. The results for the multiple trials are presented as mean ± standard error. Statistical analyses were performed using the Student’s t-test or analysis of variance, as appropriate. Statistical significance was set at *p* < 0.05. Prism software (GraphPad) was used to plot dose–response curves and calculate IC_50_ values.

## 3 Results

### 3.1 Identification of vitekwangin B from *Vitex trifolia* fruits

In previous studies, we conducted phytochemical investigations of the *Vitex* genus, including *V. limonifolia* and *V. trifolia*. These investigations led to the isolation of various compounds, including diterpenoids, flavonoids, triterpenes, and ecdysteroids (Thoa et al., 2017; Ban et al., 2018). In this study, we isolated a lignan named vitekwangin B: 4-(3,4-dimethoxyphenyl)-6-hydroxy-3-(hydroxy-methyl)-5-methoxy-3,4-dihydronaphthalene-2-carbaldehyde. We also extensively determined its chemical structure using HR-ESI-MS and NMR spectra and compared the NMR data with those reported in the literature (Supplementary Table S1A; Supplementary Figures S1–S4, Shen et al., 2019).

### 3.2 Cell-based high-throughput screening for the identification of a novel natural compound that downregulates ANO1 channel

**FIGURE 1:**
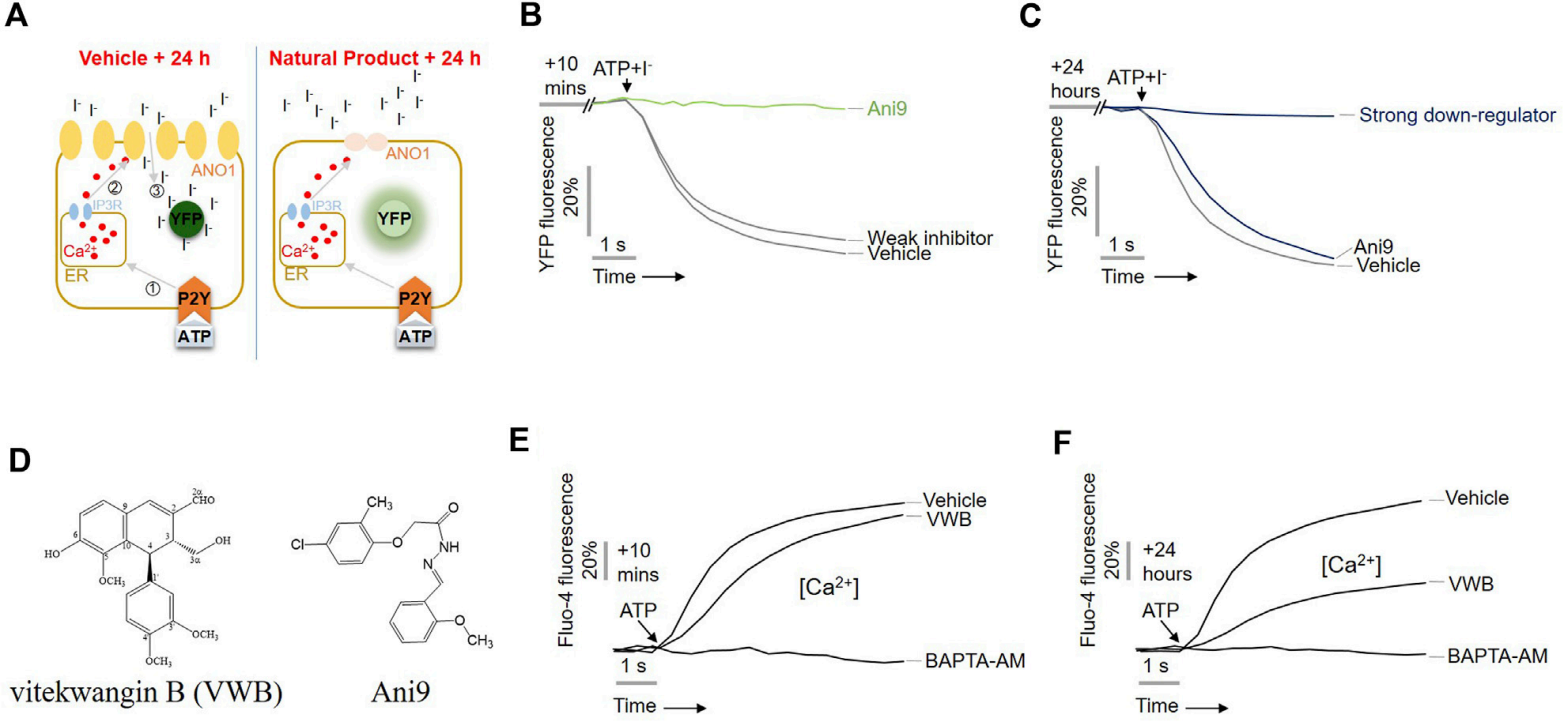
Identification of vitekwangin B using yellow fluorescent protein (YFP)-based high-throughput screening. **(A)** Schematic representation of the cell-based YFP-reduction assay. Activation of ATP-induced P2Y receptor increases calcium release from the endoplasmic reticulum, thereby activating ANO1 channel, resulting in an influx of iodide that quenches YFP. **(B,C)** YFP fluorescence levels corresponding to the inhibitory effect of the compounds Ani9 and vitekwangin B in cells treated for 10 min or 24 h. **(D)** Chemical structure of vitekwangin B and Ani9. **(E,F)** Intracellular Ca^2+^ levels were measured using Fluo-4 NW in CHO-K1 cells treated with 1 μM vitekwangin B for 20 min or 24 h and then with 100 μM ATP (mean ± SD, *n* = 5). ER, endoplasmic reticulum; VWB, vitekwangin B. ***p < 0.001, Student’s unpaired t-test.

The downregulation of ANO1 by the substances extracted from *V. negundo* was previously evaluated using a modified cell-based high-throughput screening system (Jeong et al., 2022). As shown in Figure 1, upon treatment of cells with ATP, intracellular calcium levels increased, leading to the flow of iodine into the cell through the ANO1 channel, which also acts as an iodide channel (Twyffels et al., 2014). Intracellular iodine binds to mutant YFP and strongly reduces its fluorescence. However, when an ANO1 inhibitor inhibited the ANO1 channel and blocked the iodine influx, no decrease in YFP fluorescence was observed (Figure 1A). To screen the substances isolated from *V. negundo*, that exhibit ANO1-downregulating effects, PC9-YFP cells were treated with *V. negundo* compounds for 10 min or 24 h.

Treatment with Ani9 for 10 min inhibited YFP reduction via ANO activation (Figure 1B), whereas treatment for 24 h does not; however, treatment with vitekwangin B for 24 h strongly inhibited YFP reduction via ANO1 activation (Figure 1C). The chemical structure of vitekwangin B is shown in Figure 1D. Since ANO1 is also activated by low calcium levels (Yang et al., 2008), we checked whether vitekwangin B inhibited intracellular calcium levels. PC9 cells were treated with 1 µM vitekwangin B for 10 min or 24 h to determine the effect of vitekwangin B on ATP-mediated increase in intracellular calcium signaling. Vitekwangin B treatment for 10 min did not inhibit the ATP-mediated increase in cytosolic calcium, but vitekwangin B treatment for 24 h inhibited ATP-induced increase in cytosolic calcium significantly (Figures 1E, F).

### 3.3 Effect of vitekwangin B on ANO1 protein levels and prediction of binding sites

**FIGURE 2:**
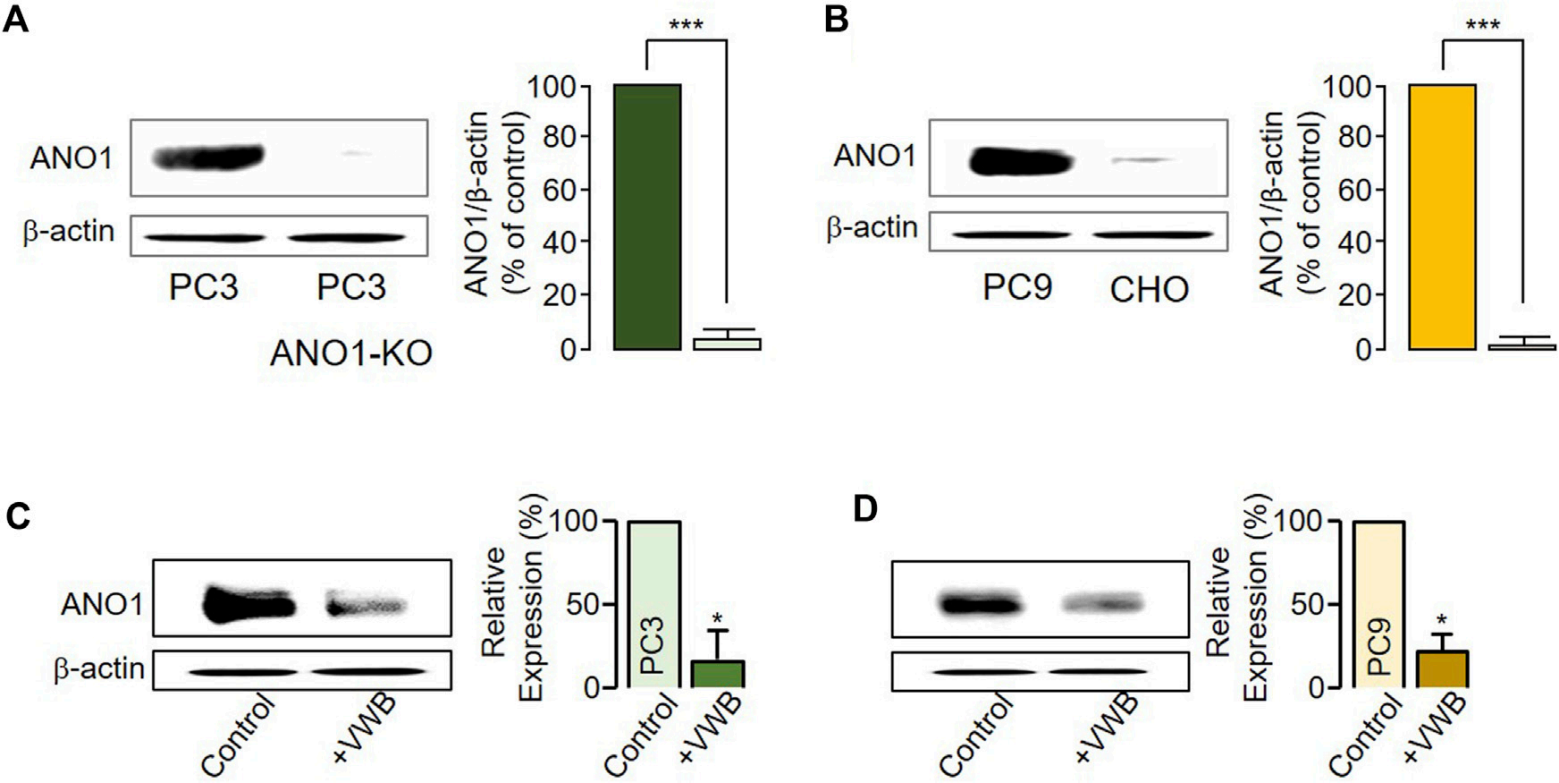
Changes in ANO1 protein expression induced by vitekwangin B. **(A)** ANO1 expression levels in PC3 and PC3 ANO1-KO cells. ANO1 protein intensities were normalized by β-actin (mean ± S.D., *n* = 5). **(B)** ANO1 expression levels in PC9 and CHO cells. ANO1 protein intensities were normalized by β-actin (mean ± S.D., *n* = 5). **(C,D)** ANO1 protein expression levels in PC3 and PC9 cells treated with 1 μM vitekwangin B. ANO1 protein intensities were normalized by β-actin (mean ± SD, *n* = 5). VWB, vitekwangin B. *p < 0.05, ***p < 0.001, Student’s unpaired t-test.

Previous studies have reported that a decrease in ANO1 protein levels can lead to decreased cancer cell viability (Seo et al., 2017; 2021; Park et al., 2023). To establish whether vitekwangin B decreased ANO1 protein levels, PC3 ANO1-KO cells were generated using the CRISPR-Cas9 system, and ANO1 protein levels in PC9 and CHO cells were verified (Figures 2A, B). After cancer cells were treated with 1 μM vitekwangin B, ANO1 protein levels were significantly decreased in PC3 and PC9 cells (Figures 2C, D).

**FIGURE 3:**
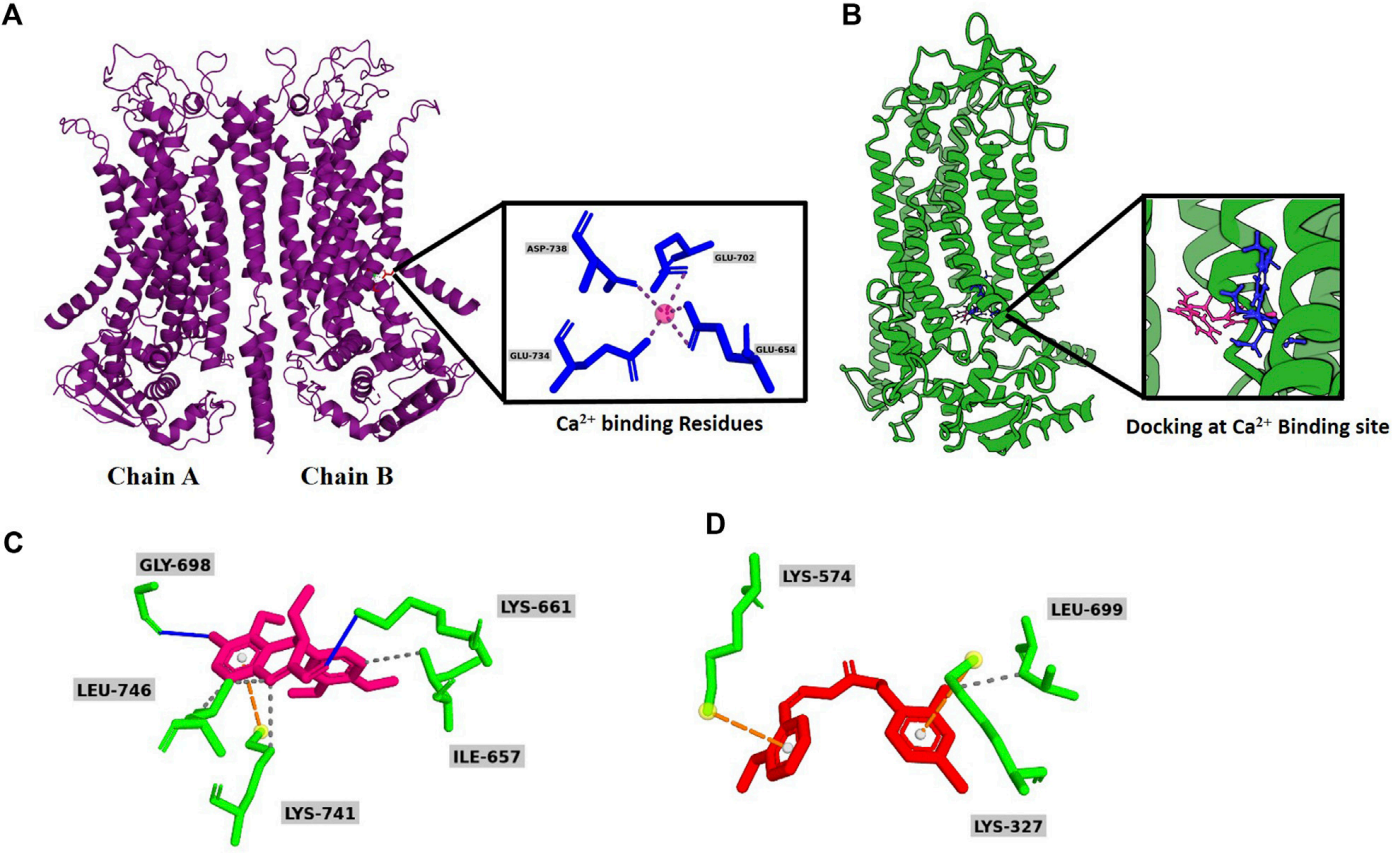
Overall graphical representation. **(A)** Secondary homodimeric structure of ANO1 (PDB ID: 5OYB) with Ca^2+^ binding site. **(B)** Molecular docking of vitekwangin B and Ani9 at Ca^2+^ binding site. **(C)** Predicted binding mode of Ani9 and **(D)** vitekwangin B with ANO1.

To verify whether vitekwangin B and Ani9 interact with the calcium-binding sites on ANO1, a cryogenic structure (5OYB) was used to perform molecular docking (Figures 3A, B). Ligand binding to ANO1 is not fully understood, but this study considered potential calcium-binding residues as ligand-binding sites based on previous mutagenesis studies. Vitekwangin B achieved a highly negative docking score of −5.091 kcal/mol, surpassing the score of Ani9 (−4.083 kcal/mol). The docking analysis and MM-GBSA scores are provided in Supplementary Table S1B. The stability of a protein–ligand complex is primarily attributed to intermolecular interactions, including hydrogen bonds, van der Waals forces, and carbon–hydrogen bonds. In this context, non-bonded interactions of protein–ligand complex were assessed, focusing on the XP docking position. Figure 3C illustrates the involvement of vitekwangin B in hydrogen and hydrophobic interactions. Specifically, vitekwangin B formed hydrogen bonds with residues Lys 661 and Gly 698 and hydrophobic interactions with residues Lys 741, Ile 657, and Leu 746. Furthermore, a pi-cation interaction was observed between vitekwangin B and the amino acid residue Lys 741. Figure 3D shows the interaction between Ani9 and ANO1, indicating hydrophobic interactions with Leu 699 and pi-cation interactions with Lys 327 and Lys 574. Notably, Ani9 did not exhibit any hydrogen bonding interactions with ANO1 residues.

### 3.4 Effect of vitekwangin B on cell viability and migration

**FIGURE 4:**
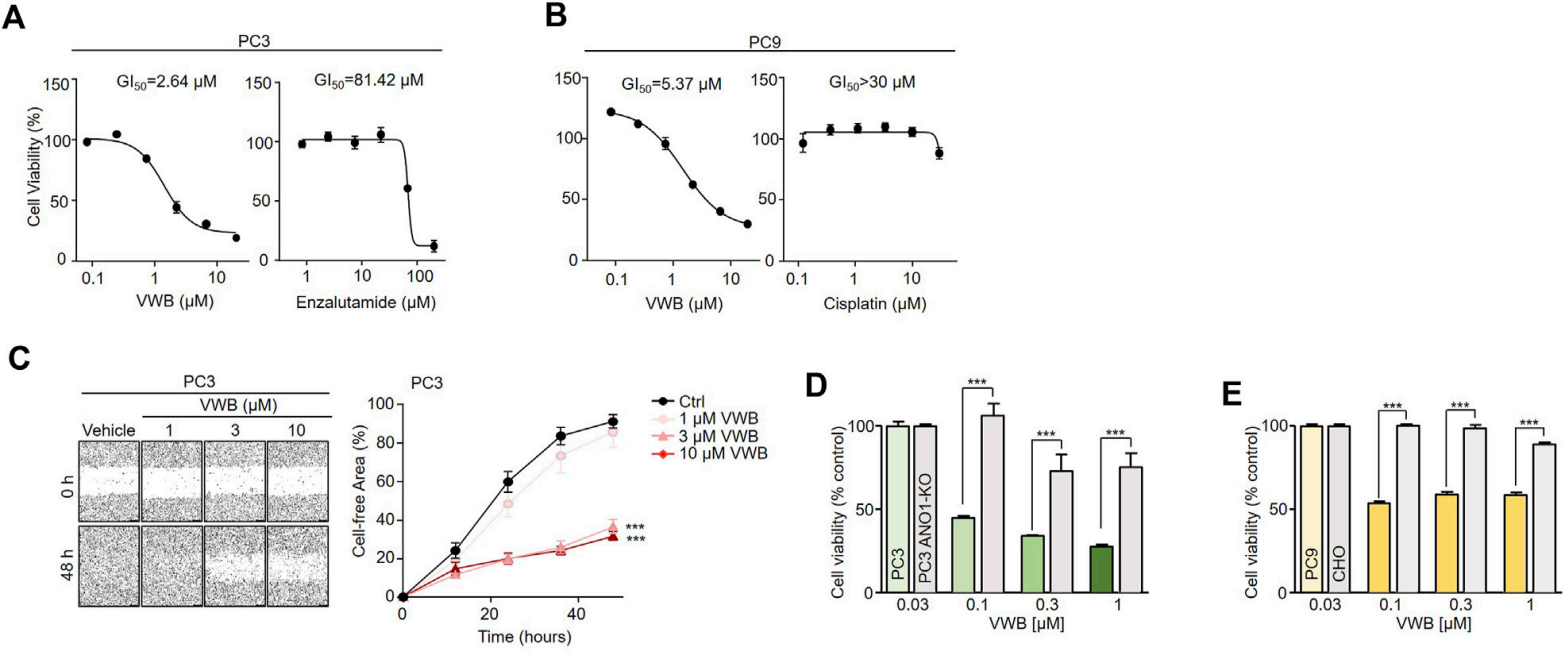
Effects of vitekwangin B on cell viability. **(A)** PC3 cells were cultured with 0.1–30 μM vitekwangin B and enzalutamide for 72 h, and cell viability was determined using CCK-8 assay. **(B)** PC9 cells were cultured with 0.1–30 μM vitekwangin B and cisplatin for 72 h, and cell viability was determined using CCK-8 assay. **(C)** Wound-healing assay was performed on ANO1-expressing PC3 cells. The cells were treated with 1, 3, and 10 μM of vitekwangin B, and representative images were acquired 0 and 64 h post-wound induction. The wound closure was quantified at 64 h. **(D,E)** Effects of the indicated concentrations of vitekwangin B on cell viability of PC3 or PC3 ANO1-KO cells and PC9 or CHO cells (mean ± SD, *n* = 5). VWB, vitekwangin B. ***p < 0.001., Student’s unpaired t-test.

Pharmacological inhibition of ANO1 inhibits the growth of various types of cancer cells (Seo et al., 2017; Seo et al., 2021; Park et al., 2023). Vitekwangin B was tested to determine whether it inhibited the growth of prostate cancer and NSCLC cells by decreasing ANO1 protein levels (Figure 4). Vitekwangin B decreased the cell viability of PC3 cells in a dose-dependent manner when incubated for 72 h, with an IC_50_ of 2.64 µM, whereas XTANDI^®^ (enzalutamide; a positive control for prostate cancer treatment) decreased cell viability with an IC_50_ of 81.42 µM. Vitekwangin B exhibited a stronger effect on PC3 cell viability, approximately 31-fold greater than that of enzalutamide (Figure 4A). Furthermore, vitekwangin B inhibited the growth of PC9 cells in a dose-dependent manner over 72 h and had an IC_50_ of 5.37 µM, whereas cisplatin (a chemotherapy drug) decreased PC9 cell viability with an IC_50_ greater than 30 µM (Figure 4B). To assess whether vitekwangin B inhibited PC3 cell migration, cells were cultured with different doses of vitekwangin B, which revealed dose-dependent inhibition of PC3 cell migration (Figure 4C). Notably, vitekwangin B showed differences in cell viability depending on ANO1 protein levels. Up to 1 µM vitekwangin B significantly inhibited the viability of ANO1-expressing cells more strongly compared to cells not expressing ANO1 and MRC-5 cells (Figures 4D, E; Supplementary Figure S5). These results indicated that vitekwangin B effectively inhibited cell viability in ANO1-expressing cells by reducing ANO1 protein levels.

### 3.5 Measurement of apoptotic effect, effect on hERG activity, and liver toxicity of vitekwangin B

**FIGURE 5:**
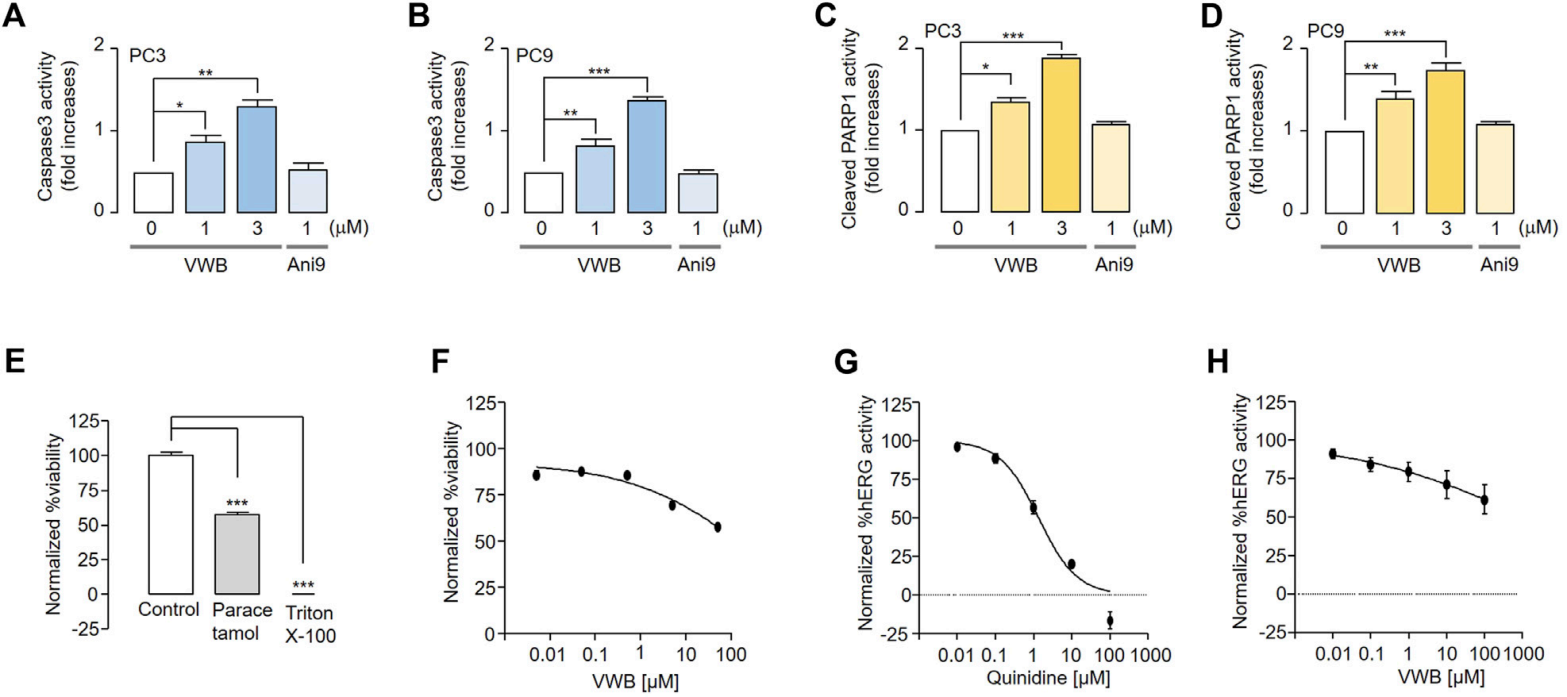
Evaluation of apoptosis and inhibition of hERG channel activity and liver cell viability induced by vitekwangin B and Ani9. **(A,B)** Caspase-3 activity in PC3 and PC9 cells cultured with vitekwangin B and Ani9 at the indicated concentrations for 24 h. **(C,D)** Cleaved PARP1 levels in PC9 cells incubated with the indicated concentrations of vitekwangin B and Ani9 for 24 h. **(E,F)** Relative viability of HepG2 cells treated with the tested compounds at five different concentrations (0.005, 0.05, 0.5, 5, and 50 μM) was determined. The viability data points were normalized while considering the maximum viability obtained with 0.5% (v/v) dimethyl sulfoxide solvent treatment as 100% viability and the minimum viability obtained with 0.01% (v/v) Triton X-100 treatment as 0% viability. **(G,H)** hERG channel activity of HEK cells treated with the tested compounds at five different concentrations (0, 0.01, 0.1, 1, 10 and 100 μM). Each data point was normalized using the baseline level of hERG channel current (mean ± SD, *n* = 3–5). VWB, vitekwangin B. *p < 0.05, **p < 0.01, ***p < 0.001, Student’s unpaired t-test.

As the pharmacological inhibition of ANO1 in various cancer cell types has been reported to cause apoptosis (Song et al., 2018; Park et al., 2023), we evaluated the apoptotic effects of vitekwangin B in PC3 and PC9 cells. Vitekwangin B significantly increased caspase-3 activity and PARP-1 cleavage, which represent two hallmarks of apoptosis (Figures 5A–D) and increased the sub-G1 population (Supplementary Figure S2). In contrast, Ani9 does not affect caspase-3 activity or PARP-1 cleavage because it does not decrease ANO1 protein levels (Seo et al., 2018). These results showed that vitekwangin B exerts anticancer effects by inducing apoptosis and reducing ANO1 protein levels.

To verify whether vitekwangin B is toxic to liver cells, HepG2 cells were treated with vitekwangin B, and their viability was measured. Vitekwangin B did not inhibit liver cell viability up to 50 μM, as compared with paracetamol (Figures 5E, F). In addition, the effects of vitekwangin B on hERG channel activity were tested in hERG channel-expressing HEK293 cells to confirm its potential applications in cancer treatment. Vitekwangin B did not inhibit hERG channel activity at concentrations up to 100 μM, and 10 μM quinidine (positive control) completely inhibited the hERG channel (Figures 5G,H).

## 4 Discussion

While ANO1 plays multifaceted roles in various physiological processes, such as chloride ion secretion and cancer development, its chemotherapeutic potential is realized through the inhibition of its function and reduction of its protein expression (Britschgi et al., 2013; Liu et al., 2021). Therefore, several ANO1 inhibitors have been developed (Zhong et al., 2021). Several research groups have attempted to develop specific ANO1 inhibitors, starting from hit to lead chemical compounds via lead optimization (Seo et al., 2018; Zhong et al., 2021) but reported that ANO1 inhibitors may have side effects such as dry mouth syndrome, lowered blood pressure, and inhibited intestinal motility because they inhibit ANO1 channel function (Britschgi et al., 2013; Bill and Gaither, 2017; Liu et al., 2021; Wang et al., 2021; Zhong et al., 2021; Jeong et al., 2022). Therefore, it is necessary to develop novel small-molecule drugs that can gradually reduce ANO1 protein levels without causing side effects in humans.

In this study, we found that vitekwangin B did not inhibit ANO1 channel function but reduced ANO1 protein levels strongly. Remarkably, treatment with vitekwangin B for 10 min did not inhibit ATP-induced calcium influx but treatment for 24 h inhibited ATP-induced increase in cytosolic calcium significantly (Figure 1).

Because vitekwangin B reduced ANO1 protein levels notably, thereby reducing cell survival, it appeared to affect intracellular calcium changes (Figure 2). In addition, this phenomenon may have occurred because vitekwangin B interacts with a known calcium-binding site in ANO1 (Figure 3). Ani9, a known ANO1 inhibitor, also interacted with the calcium binding site on ANO1; however, vitekwangin B appeared to have better hydrogen bond binding than Ani9, and other compounds appeared to interact with the ANO1 protein (Figure 3). ANO1 is a homodimer channel harboring two pores that function independently, with one pore in each monomer. Each pore is activated by voltage-dependent binding of two intracellular calcium ions to a high-affinity binding site. In addition, the binding of phosphatidylinositol 4,5-bisphosphate to sites scattered throughout the cytosolic side of the protein aids in calcium activation process (Arreola et al., 2022). Moreover, Shi et al. (2021) demonstrated that theaflavin plays an important role in the pore blockade of ANO1 through mutagenesis experiments and inhibits viability in lung adenocarcinoma cells. However, because of the structural diversity of compounds and binding pockets on ANO1, it will be necessary to measure the binding affinity of vitekwangin B and ANO1 accurately through mutation experiments in the future. However, it is important to note that vitekwangin B reduced protein levels without inhibiting the function of the ANO1 channel, thereby contributing to reduced cell survival (Figure 4).

According to our experimental findings, the reduction in the cell viability of cells treated with drugs for prostate cancer and general anticancer drugs were less than that observed upon treatment with vitekwangin B (Figure 4). Thus far, no synthetic ANO1 inhibitors have entered clinical trials, and no substances have been developed with *in vitro* safety and excellent *in vivo* effectiveness using animal models (Namkung et al., 2011; Britschgi et al., 2013; Bill and Gaither, 2017; Seo et al., 2017; 2018; 2021; Liu et al., 2021; Wang et al., 2021; Zhong et al., 2021; Jeong et al., 2022). Therefore, natural products that reduce the ANO1 protein levels notably are novel and could be a breakthrough in the development of ANO1 inhibitors.

We discovered that vitekwangin B suppresses cell survival by notably reducing ANO1 protein levels and that its effect on cell viability in ANO1-KO cells was relatively weak (Figures 2, 4). In addition, vitekwangin B induced apoptosis, a characteristic feature of anticancer drugs (Supplementary Figure S6). It was confirmed that Ani9, which only inhibits the function of ANO1 channel, does not cause apoptosis (Figure 5). Therefore, it can be inferred that vitekwangin B reduced cell survival via apoptosis. Considering that it has little effect on hepatocellular toxicity and hERG channel function, which is important for the heartbeat, vitekwangin B can be developed as an anticancer drug. During the initial stages of drug development, it is crucial to assess cardiotoxicity and hepatotoxicity. However, it is equally important to evaluate additional pharmacokinetic factors such as absorption, distribution, metabolism, excretion, and toxicity to determine the properties and toxicity of a substance in animal models prior to its application in the human body.

In summary, our cell-based high throughput screening led us to discover the anticancer potential of vitekwangin B, which significantly reduced ANO1 protein levels, diminished cell viability, and triggered apoptosis. Vitekwangin B, a natural product with no hepatocellular toxicity or adverse effects on cardiac channels, holds promise as a valuable pharmacological tool for ANO1 inhibitor research. Furthermore, its potential application in the treatment of prostate and lung cancers is noteworthy, particularly considering the common resistance of these cancers to existing anticancer drugs. Further investigations are warranted to determine whether vitekwangin B, a relatively nontoxic natural product, can help overcome resistance to current anticancer medications in these specific cancer types.

## Data Availability

The datasets presented in this study can be found in online repositories. The names of the repository/repositories and accession number(s) can be found in the article/Supplementary Material.
