# Supplementary material for: Discovery of a novel natural compound, vitekwangin B, with ANO1 protein reduction properties and anticancer potential
Source: Front Pharmacol. 2024 Apr 3;15:1382787. doi: 10.3389/fphar.2024.1382787 (PMC11041392; doi:10.3389/fphar.2024.1382787)
Supplement: Supplementary file 1 [file DataSheet1.pdf]

Supplementary Material

1 Supplementary Tables

Supplementary Table S1A. ^{1}H and ^{13}C NMR data of vitekwangin B

	δ_C [a,b]	δ_H (J = Hz) [a,c]
1	149.0	7.49 (s)
2	135.8	-
2α	194.4	9.47 (s)
3	43.5	3.23 (dd, 10.2, 4.8)
3α	62.8	3.50 (dd, 10.8, 4.8)<br>3.11 (dd, 10.8, 10.2)
4	38.5	4.90 (s)
5	147.8	-
6	155.5	-
7	116.7	6.90 (d, 8.4)
8	127.8	7.19 (d, 8.4)
9	125.8	-
10	134.2	-
1′	138.1	-
2′	113.0	6.75 (d, 1.8)
3′	150.3	-
4′	149.0	-
5′	112.9	6.73 (d. 8.4)
6′	120.9	6.45 (dd, 8.4, 1.8)
5-OMe	60.9	3.56 (s)
3′-OMe	56.5	3.74 (s)
4′-OMe	56.3	3.74 (s)

a) MeOD, b) 125 MHz, c) 500 MHz.

Supplementary Table S1B. Docking and binding free energy (MM-GBSA) scores for the selected compounds.

Compound Name	ANO1 PDB ID: 5OYB	
	XP docking score (kcal/mol)	MM-GBSA (kcal/mol)
Ani9	-4.083	-33.08
Vitedoin A	-5.091	-38.52

Supplementary Table S1C. Docking interaction patterns of ANO1 (5OYB) with selected compounds.

Compound Name	Hydrogen bond (length in Å)	Hydrophobic bond (length in Å)	π-Cation
Ani9		Leu^{699} (3.71)	Lys^{327} (4.41)<br>Lys^{574} (3.97)
Vitedoin A	Lys^{661} (1.97)<br>Gly^{698} (1.86)	Lys^{741} (3.79)<br>Ile^{657} (3.81)<br>Leu^{746} (3.67)<br>Leu^{746} (3.56)	Lys^{741} (4.19)

2 Supplementary Figures

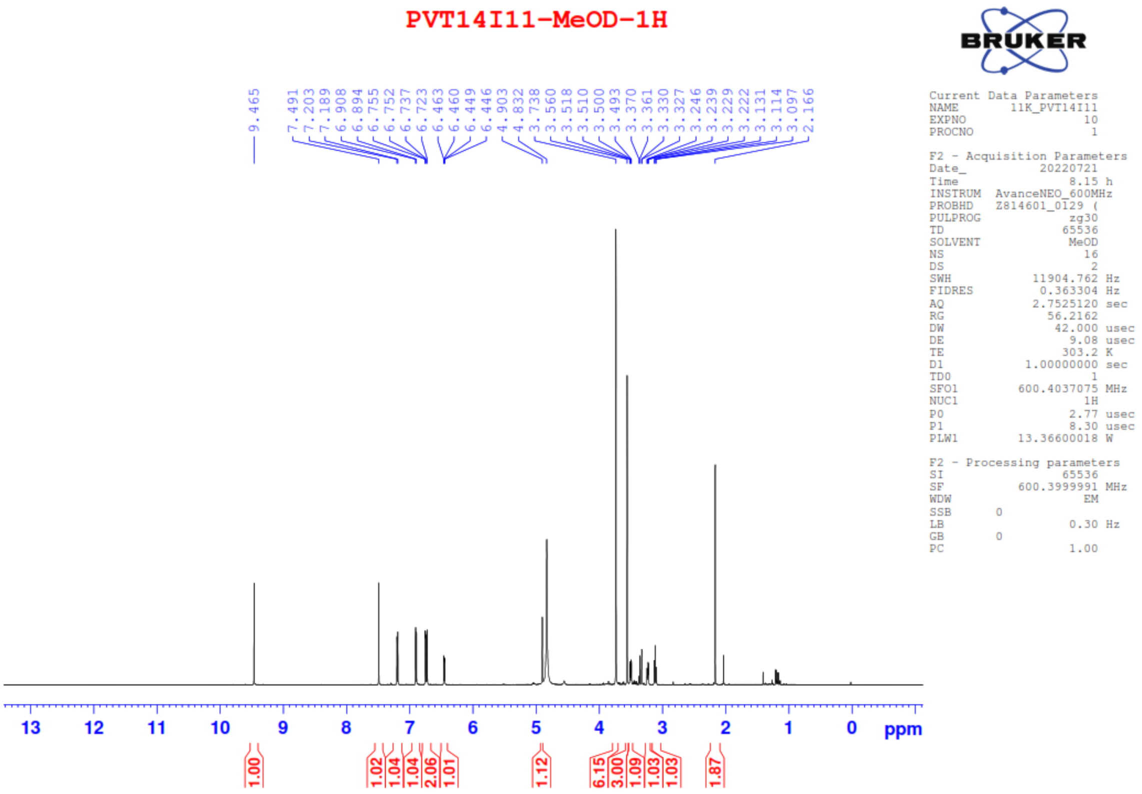

Supplementary Figure S1. ^{1}H NMR spectrum of vitekwangin B.

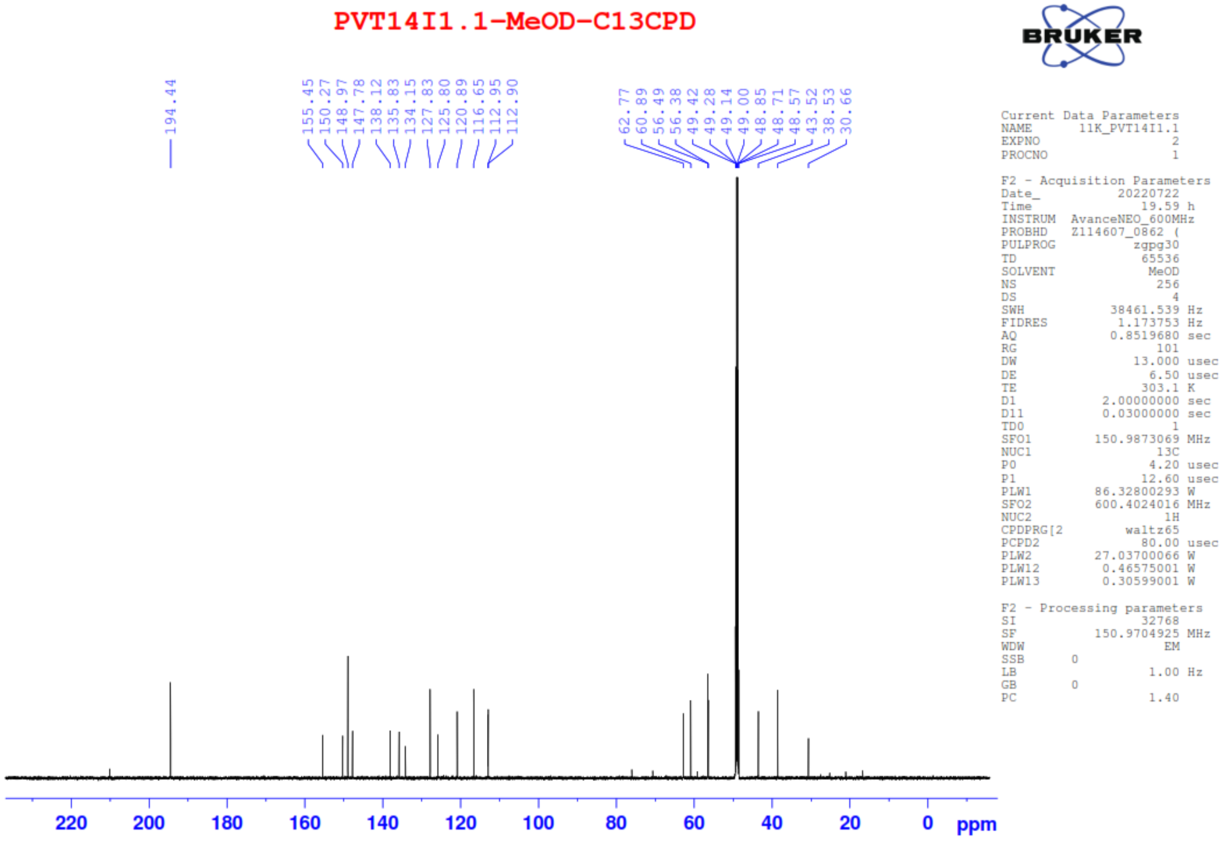

Supplementary Figure S2. ^{13}C NMR spectrum of vitekwangin B.

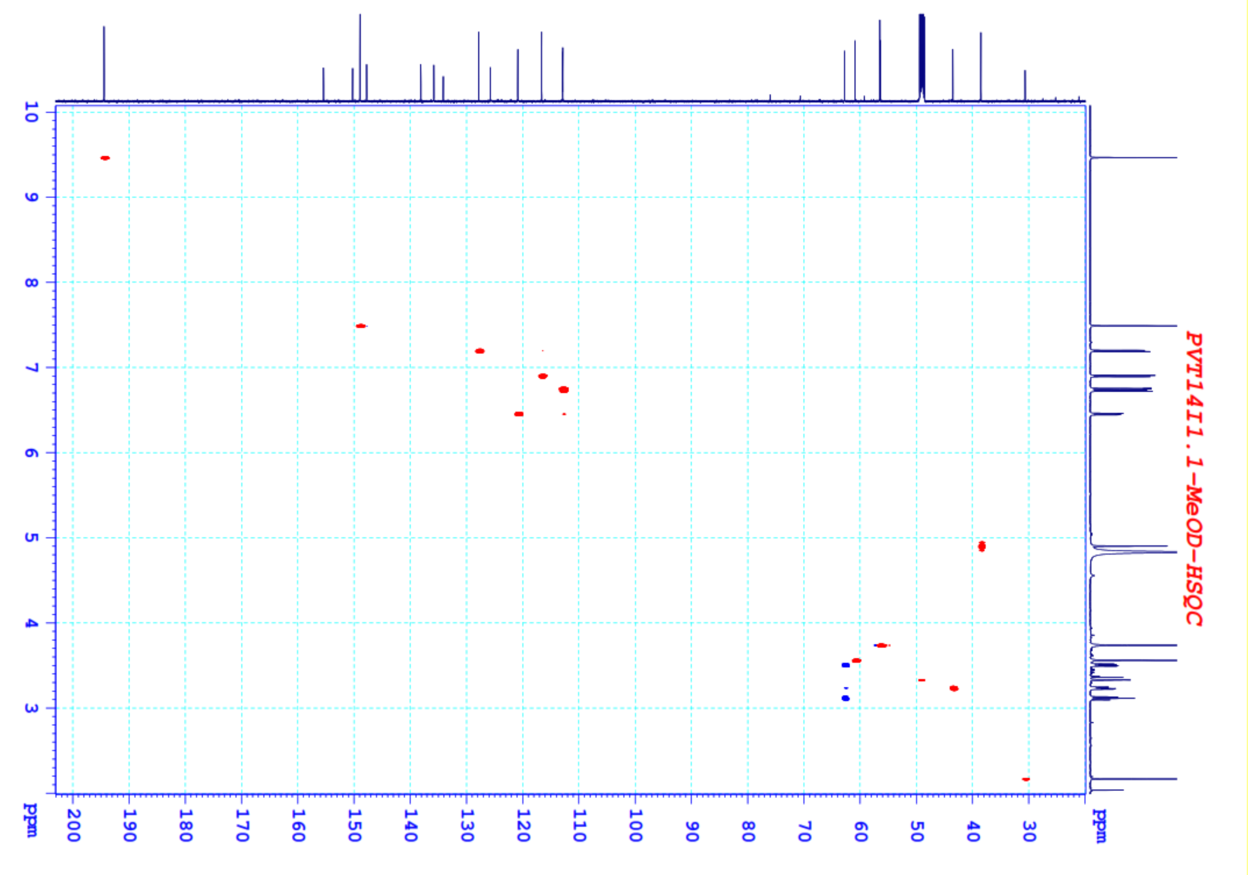

Supplementary Figure S3. HSQC spectrum of vitekwangin B.

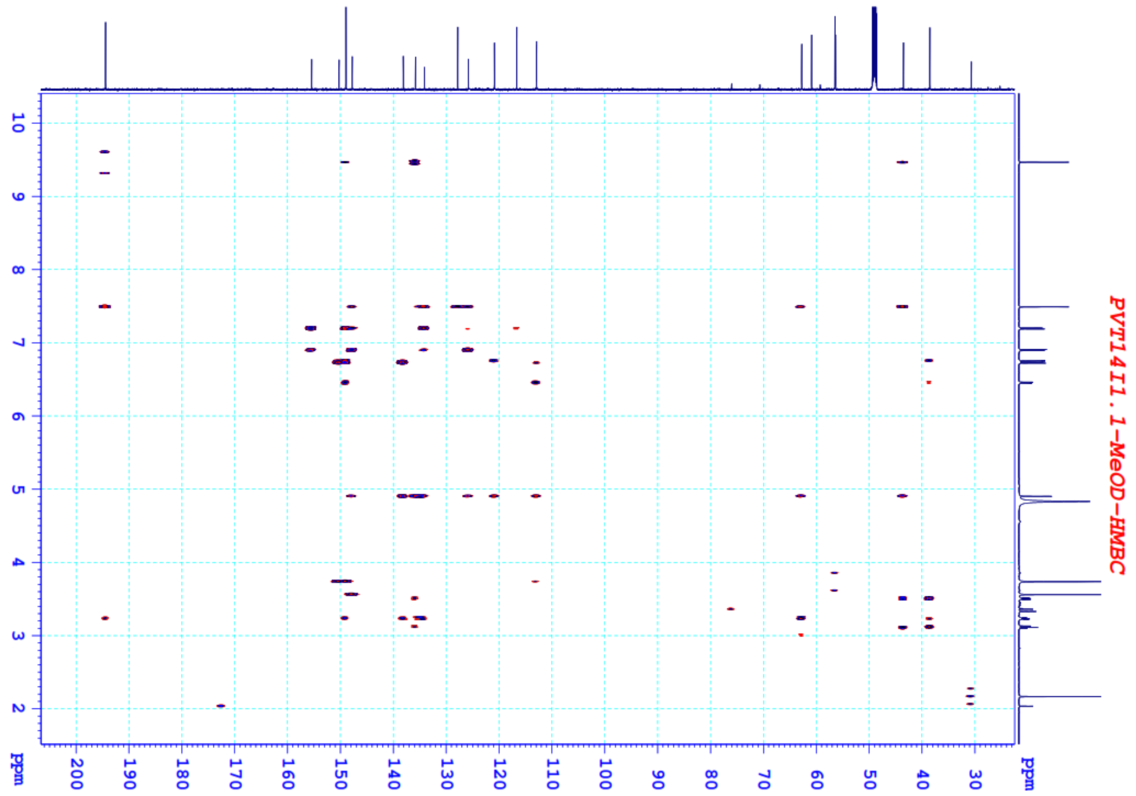

Supplementary Figure S4. HMBC spectrum of vitekwangin B.

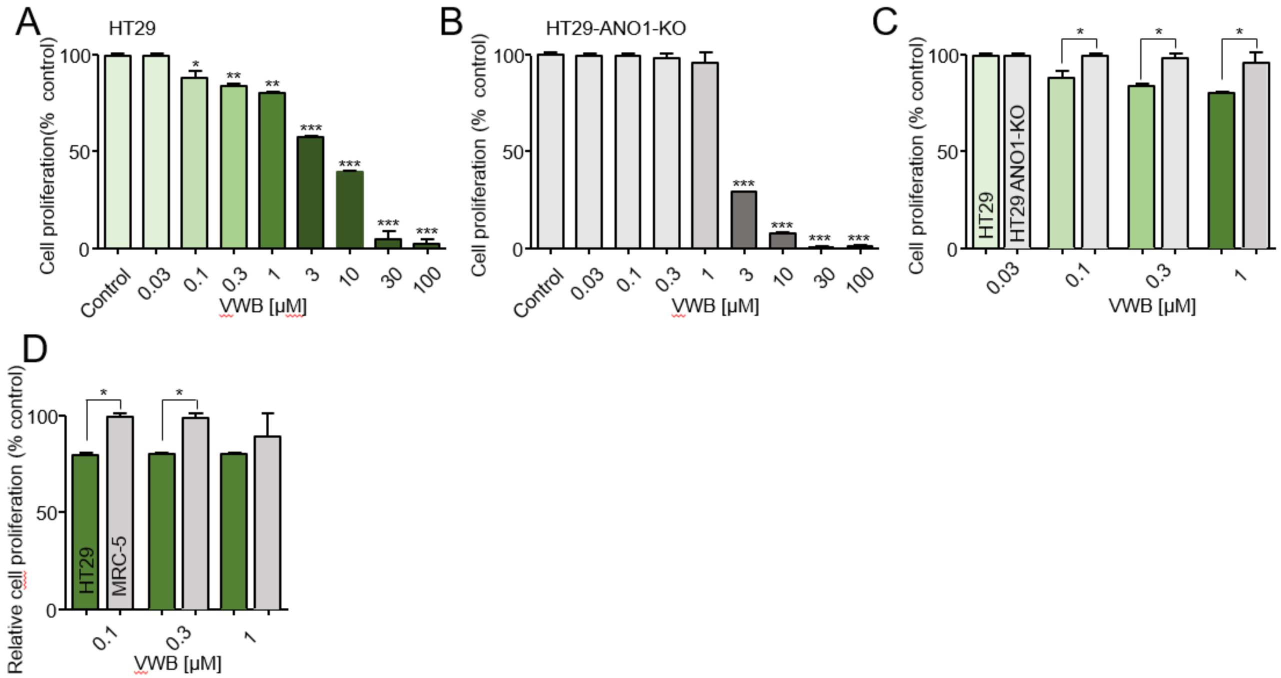

Supplementary Figure S5. Cell proliferation of HT29, HT29ANO1KO, and MRC-5 cells treated with vitekwangin B.

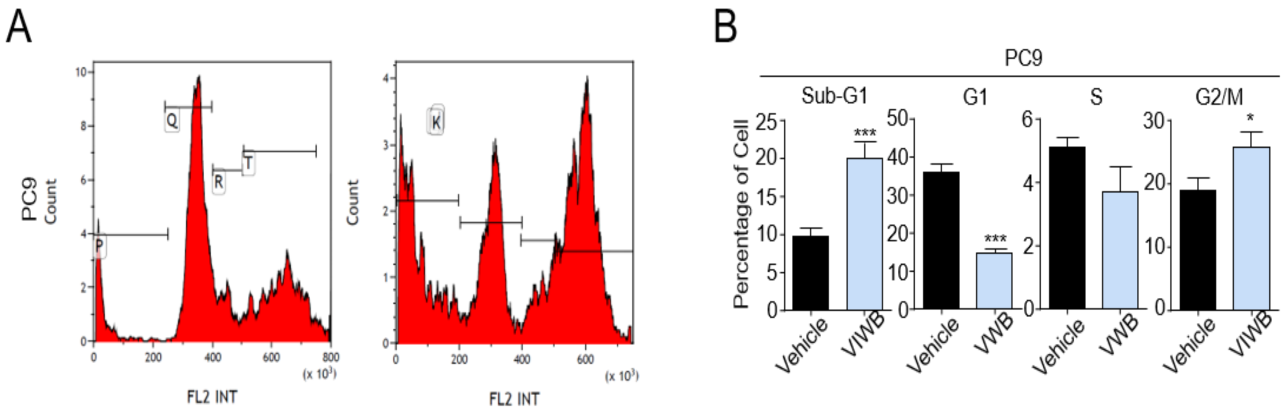

Supplementary Figure S6. Percentage of cells during the cell cycle in PC9 cells treated with the vehicle and vitekwangin B.

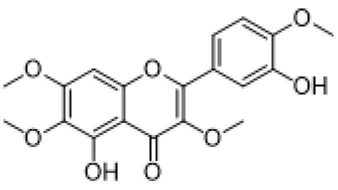

Casticin

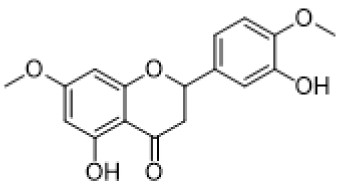

Persicogenin

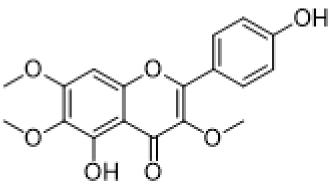

Penduletin

Supplementary Figure S7. Percentage of cells on cell cycle by vitekwangin B in PC9 cells.
